# Supplementary material for: Reconciling Mining with the Conservation of Cave Biodiversity: A Quantitative Baseline to Help Establish Conservation Priorities
Source: PLoS One. 2016 Dec 20;11(12):e0168348. doi: 10.1371/journal.pone.0168348 (PMC5173368; doi:10.1371/journal.pone.0168348)
Supplement: S1 Dataset — (ZIP) [file pone.0168348.s002.zip › Taxa/Serra Norte/SN_2013/N4WS/Lista N4WS-66.pdf]

CAVIDADE N4WS-0066

Classe	Ordem	Fam/Outros	Gên/Outros	Espécie	S-U	U-S	Zon.
Arachnida	Araneae	Filistatidae		sp.1	X	X	E
Arachnida	Araneae	Pholcidae	*Mesabolivar*	*aurantiacus*		X	E
Arachnida	Araneae	Pholcidae	*Mesabolivar*	*eberhard*	X		E
Arachnida	Araneae	Uloboridae	*Uloborus*	sp.1	X		E
Insecta	Coleoptera	Tenebrionidae		jovem		X	E
Insecta	Diptera	Ceratopogonidae		jovem	X		E
Insecta	Diptera	Dolichopodidae		sp.	X		E
Insecta	Diptera	Tipulidae	Tipulinae	sp.	X		E
Insecta	Heteroptera	Reduviidae	Reduviinae	jovem	5		E
Insecta	Homoptera	Cixiidae		sp.3	X		E
Insecta	Isoptera	Termitidae	*Cortaritermes*	*silvestrii*	X		E
Insecta	Isoptera	Termitidae	*Nasutitermes*	sp.		X	E
Insecta	Lepidoptera	Noctuoidea		sp.1	2		E
Insecta	Neuroptera	Myrmeleontidae		jovem	500		E
Insecta	Orthoptera	Phalangopsidae	*Paraclodes*	sp.	3		E
Insecta	Psocoptera	Psocomorpha		jovem		X	E
Amphibia	Anura	Strabomantidae	*Pristimantis*	cf. *fenestratus*	2		E
